# Supplementary material for: Isoprostanoid Profiling of Marine Microalgae
Source: Biomolecules. 2020 Jul 18;10(7):1073. doi: 10.3390/biom10071073 (PMC7407139; doi:10.3390/biom10071073)
Supplement: Supplementary file 1 [file biomolecules-10-01073-s001.zip › biomolecules-843401-supplementary/supplementary data last/Figures S1, 2.docx]

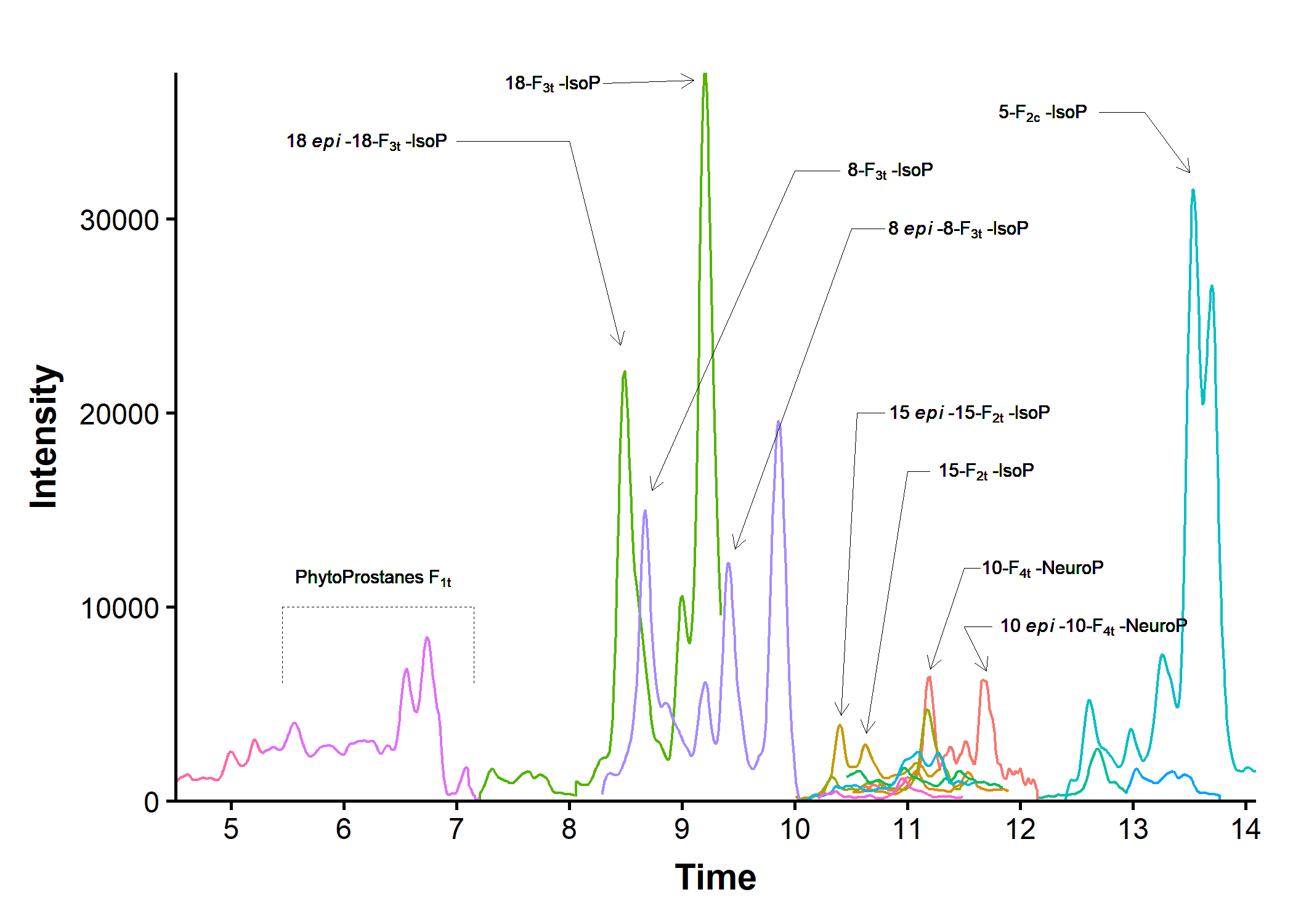


**Figure S1.** Chromatogram of selected reaction monitoring (SRM) of metabolites detected in *Chaetoceros gracilis.*


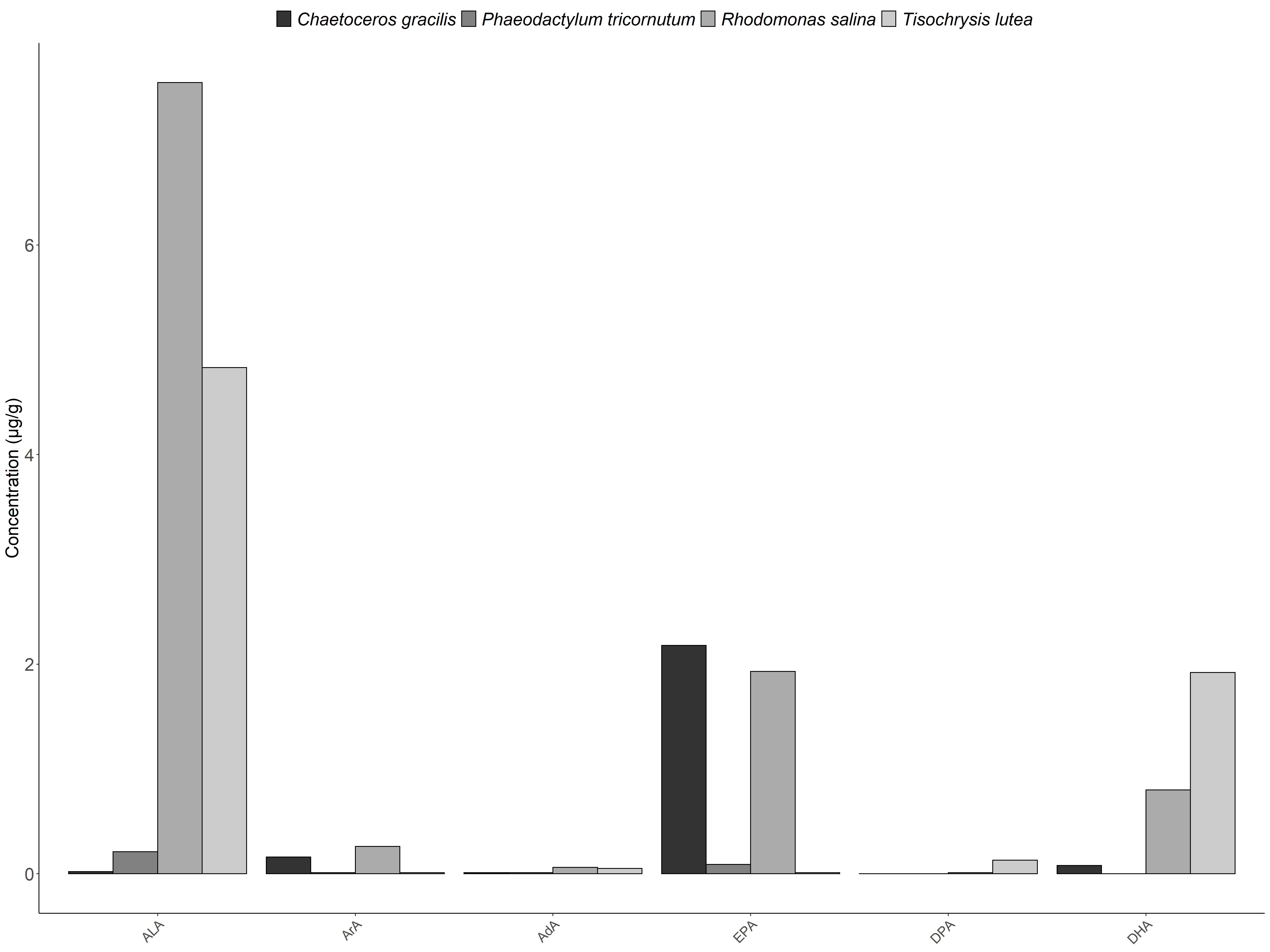


**Figure S2.** Distribution of oxidized metabolites (sum of concentrations) classified according to the original PUFAs in the four species studied.
