# Supplementary material for: Isoprostanoid Profiling of Marine Microalgae
Source: Biomolecules. 2020 Jul 18;10(7):1073. doi: 10.3390/biom10071073 (PMC7407139; doi:10.3390/biom10071073)
Supplement: Supplementary file 1 [file biomolecules-10-01073-s001.zip › biomolecules-843401-supplementary/supplementary data last/Table S1.pdf]

Analyte	Retention Time (min)	m/z Precusur Ion	m/z Product Ion	DP (volts)	EP (volts)	CE (volts)	CXP (volts)
Alpha-Linolenic acid							
16-F_{1t}-PhytoP	6,22	327,2	283	-100	-5	-27	-15
			251	-100	-5	-27	-15
16-*ep* i-16-F_{1t}-PhytoP	5,73	327,2	283	-100	-5	-27	-15
			251	-100	-5	-27	-15
9-F_{1t}-PhytoP	5,93	327,2	283	-100	-5	-27	-15
			171	-100	-5	-27	-15
9-*epi*-9-F_{1t}-PhytoP	6,44	327,2	283	-100	-5	-27	-15
			171	-100	-5	-27	-15
ent-16-B_1-PhytoP	10,62	307,2	235	-130	-5	-28	-18
			223	-130	-5	-28	-18
ent-9-L_1-PhytoP	10,79	307,2	185	-152	-9	-28	-10
			109	-152	-9	-29	-6
16(*RS*)-16-A_1-PhytoP	11,26	307,2	289	-115	-9	-15	-17
			249	-115	-9	-22	-18
ent-9-*epi*-9-D_{1t}-PhytoP	7,63	325,0	289	-121	-10	-22	-18
			123	-121	-10	-34	-7
			135	-121	-10	-29	-7
ent-9-D_{1t}-PhytoP	8,43	325,0	289	-121	-10	-22	-8
			123	-121	-10	-34	-7
			135	-121	-10	-29	-7
ent-16(*RS*)-9-*epi*-ST-Δ^{14}-10-PhytoF	5,00	343,2	209	-151	-8	-31	-7
			83	-151	-8	-37	-7
ent-9(*RS*)-12-*epi*-ST-Δ^{10}-13-PhytoF	5,00	343,2	237	-161	-7	-33	-27
			87	-161	-7	-36	-27
ent-16(*RS*)-13-*epi*-ST-Δ^{14}-9-PhytoF	4,89	343,2	201	-158	-6	-35	-12
			127	-158	-6	-43	-7
C19-PhytoP	9,10	241,3	297	-149	-7	-30	-16
			279	-149	-7	-32	-14
Arachidonic acid							
15-A_2-IsoP	15,21	333,2	271	-141	-8	-17	-17
			189	-141	-8	-23	-17
15-F_{2t}-IsoP	10,50	353,2	193	-147	-5	-32	-10
			247	-100	-10	-37	-27
15-*ep* i-15-F_{2t}-IsoP	10,30	353,2	193	-147	-5	-32	-10
			247	-100	-10	-37	-27
5(*RS*)-5-F_{2t}-IsoP	10,91	353,2	115	-203	-5	-25	-37
			309	-203	-5	-25	-37
5-F_{2c}-IsoP	13,40	353,2	115	-149	-5	-29	-12
			309	-149	-5	-27	-20
C21-15-F_{2t}-IsoP	12,50	367,0	193	-174	-3	-34	-27
			261	-174	-3	-34	-27
d_4-15-F_{2t}-IsoP	10,50	257,2	197	-65	-6	-35	-12
			251	-65	-6	-35	-12
			213	-100	-10	-37	-27
Adrenic acid							
ent-7(*RS*)-7-F_{2t}-dihomo-IsoP	13,43	381,3	143	-161	-6	-32	-8
			327	-161	-6	-28	-18
7(*RS*)-ST-Δ^{8}-11-dihomo-IsoF	13,28	397,4	201	-185	-5	-34	-11
			183	-185	-5	-36	-10
Eicosapentaenoic acid							
18-*epi*-18-F_{3t}-IsoP	8,37	351,2	289	-167	-7	-27	-16
			249	-167	-7	-29	-13
18-F_{3t}-IsoP	9,06	351,2	289	-167	-7	-27	-16
			249	-167	-7	-29	-13
5-*epi*-5-F_{3t}-IsoP	9,14	351,2	307	-140	-3	-24	-22
			115	-140	-3	-27	-7
5-F_{3t}-IsoP	8,91	351,2	307	-140	-3	-24	-22
			115	-140	-3	-27	-7
8-*epi*-8-F_{3t}-IsoP	9,29	351,3	127	-151	-8	-31	-7
			155	-151	-8	-27	-10
8-F_{3t}-IsoP	8,56	351,3	127	-151	-8	-31	-7
			155	-151	-8	-27	-10
Docosapentaenoic acid							
4-F_{3t}-NeuroP	14,84	379,2	101	-174	-4	-29	-8
			273	-174	-4	-32	-12
Docosahexaenoic acid							
4(*RS*)-4-F_{4t}-NeuroP	12,50	377,2	101	-161	-5	-28	-7
			217	-161	-5	-27	-16
10-*epi*-F_{4t}-NeuroP	11,46	377,2	153	-149	-4	-27	-14
			110	-149	-4	-27	-8
10-F_{4t}-NeuroP	11,02	377,2	153	-149	-4	-27	-8
			110	-149	-4	-27	-8
13(*RS*)-13-F_{4t}-NeuroP	10,8 ; 11,18	377,2	193	-149	-4	-27	-8
14(*RS*)-14-F_{4t}-NeuroP	11,65	377,2	179	-166	-5	-28	-10
			205	-166	-5	-29	-12
20-*epi*-20-F_{4t}-NeuroP	10,80	377,2	315	-164	-3	-26	-18
			323	-164	-3	-26	-18
			259	-164	-3	-27	-14
20-F_{4t}-NeuroP	11,26	377,2	315	-164	-3	-26	-18
			323	-164	-3	-26	-18
			259	-164	-3	-27	-14
d_4-10-*epi*-10-F_{4t}-NeuroP	11,00	381,2	157	-155	-5	-27	-11
			110	-155	-5	-32	-6
