# Supplementary material for: Isoprostanoid Profiling of Marine Microalgae
Source: Biomolecules. 2020 Jul 18;10(7):1073. doi: 10.3390/biom10071073 (PMC7407139; doi:10.3390/biom10071073)
Supplement: Supplementary file 1 [file biomolecules-10-01073-s001.zip › biomolecules-843401-supplementary/supplementary data last/Table S2.pdf]

Analyte	**Linear regression**	**R^2**
Alpha-Linolenic acid		
16-F_{1t}-PhytoP	y = 1,59081 x	0,998
16-*ep*i-16-F_{1t}-PhytoP	y = 1,60226 x	0,996
9-F_{1t}-PhytoP	y = 1,20477 x	0,996
9-*epi*-9-F_{1t}-PhytoP	y = 1,07008 x	0,998
ent-16-B_1-PhytoP	y = 2,23001 x	0,998
ent-9-L_1-PhytoP	y = 3,28989 x	0,999
16(*RS*)-16-A_1-PhytoP	y = 1,81896 x	0,999
ent-9-*epi*-9-D_{1t}-PhytoP	y = 3,50478x	0,997
ent-9-D_{1t}-PhytoP	y = 2,94021x	0,999
ent-16(*RS*)-9-*epi*-ST-Δ^{14}-10-PhytoF	y = 2,51294x	0,995
ent-9(*RS*)-12-*epi*-ST-Δ^{10}-13-PhytoF	y = 1,12189 x	0,996
ent-16(*RS*)-13-*epi*-ST-Δ^{14}-9-PhytoF	y = 3,10290 x	0,996
Arachidonic acid		
15-A_2-IsoP	y = 3,55777 x	0,993
15-F_{2t}-IsoP	y = 3,51033 x	0,999
15-*ep*i-15-F_{2t}-IsoP	y = 3,61580 x	0,995
5(*RS*)-5-F_{2t}-IsoP	y = 1,87935 x	0,995
5-F_{2c}-IsoP	y = 4,05767 x	0,997
Adrenic acid		
ent-7(*RS*)-7-F_{2t}-dihomo-IsoP	y = 2,38407 x	0,984
7(*RS*)-ST-Δ^{8}-11-dihomo-IsoF	y = 1,05772 x	0,986
Eicosapentaenoic acid		
18-*epi*-18-F_{3t}-IsoP	y = 0,88080 x	0,988
18-F_{3t}-IsoP	y = 2,35170 x	0,991
5-*epi*-5-F_{3t}-IsoP	y = 1,54771 x	0,985
5-F_{3t}-IsoP	y = 0,55624 x	0,977
8-*epi*-8-F_{3t}-IsoP	y = 5,14933 x	0,998
8-F_{3t}-IsoP	y = 7,11727 x	0,994
Docosapentaenoic acid		
4-F_{3t}-NeuroP	y = 0,68245 x	0,990
Docosahexaenoic acid		
4(*RS*)-4-F_{4t}-NeuroP	y = 2,15917 x	0,986
10-*epi*-F_{4t}-NeuroP	y = 3,04646 x	0,997
10-F_{4t}-NeuroP	y = 5,30507 x	0,997
13(*RS*)-13-F_{4t}-NeuroP	y = 2,03321 x	0,995
	y = 1,00581 x	0,993
14(*RS*)-14-F_{4t}-NeuroP	y = 0,31517 x	0,997
20-*epi*-20-F_{4t}-NeuroP	y = 1,16434 x	0,985
20-F_{4t}-NeuroP	y = 0,94180 x	0,991
